# Breast cancer brain metastases: evidence for neuronal-like adaptation in a ‘breast-to-brain’ transition?

**DOI:** 10.1186/bcr3651

**Published:** 2014-05-06

**Authors:** Amanda ED Van Swearingen, Marni B Siegel, Carey K Anders

**Affiliations:** 1 Lineberger Comprehensive Cancer Center, University of North Carolina at Chapel Hill, 450 West Drive, Campus Box #7295, Chapel Hill, NC 27599, USA; 2 Department of Genetics, University of North Carolina at Chapel Hill, 120 Mason Farm Road, Campus Box #7264, Chapel Hill, NC 27599, USA; 3 Department of Medicine, Division of Hematology/Oncology, University of North Carolina at Chapel Hill, 170 Manning Drive, POB Room 3119, Campus Box #7305, Chapel Hill, NC 27599, USA

## Abstract

Brain metastases remain a significant challenge in the treatment of breast cancer patients due to the unique environment posed by the central nervous system. A better understanding of the biology of breast cancer cells that have metastasized to the brain is required to develop improved therapies. A recent *Proceedings of the National Academy of Sciences* article demonstrates that breast cancer cells in the brain microenvironment express γ-aminobutyric acid (GABA)-related genes, enabling them to utilize GABA as an oncometabolite, thus gaining a proliferative advantage. In this viewpoint, we highlight these findings and their potential impact on the treatment of breast cancer brain metastases.

## Breast cancer brain metastases represent a clinically unmet need and are poorly understood

Breast cancer brain metastases (BCBMs) are an increasingly common occurrence and portend a poor prognosis [1]. Treatment of BCBMs represents an unmet medical need as there are currently no approved therapies aside from surgical resection and cranial radiation [2]. Limited systemic treatment options for BCBMs are due, in part, to the unique challenges posed by the neural environment. Protection afforded by the blood-brain barrier produces an immune-privileged sanctuary and prevents many systemic therapies from penetrating the brain, affording cancer cells unique opportunities to flourish within the neural environment. The added complication of drug delivery to BCBMs coupled with our poor understanding of the factors unique to BCBM growth, metabolism, and stromal interactions continues to hinder the development of effective therapeutic strategies.

Two major questions regarding BCBMs are (1) what factors optimize BCBM cells to seed and survive in the brain microenvironment, and (2) whether the factors are present in the primary breast cancer cells or are acquired during metastasis. Among the breast cancer subtypes, HER2-enriched (HER2+) and triple-negative primary tumors show higher rates of brain relapse relative to hormone receptor-positive tumors [3,4], suggesting that some innate feature(s) of primary tumor cells may dictate the development of brain metastases. Recent literature suggests that, prior to reaching the brain, some metastatic primary breast cancer cells already express proteins that are essential for the establishment of brain metastases, including serpins [5], matrix metalloproteases [6,7], and αB-crystallin [8]. Once in the brain, recent studies have shown BCBM cells transform the surrounding glia and neurons to create a more permissive microenvironment [9-11]. Of note, tumor-activated astrocytes have been shown to play a chemo-protective role for brain metastases [9] and enhance cancer cell proliferation and survival [6,12]. Targeting reactive astrocytes in the tumor microenvironment yields therapeutic benefit *in vivo* by decreasing cancer cell seeding growth in the brain [13,14]. Thus, current evidence suggests that BCBMs are the result of both primed breast cancer cells as well as adaptation to and alteration of the brain-tumor microenvironment. Better understanding of the brain microenvironment and its interactions with breast cancer cells are critical for the development of improved treatments for BCBMs.

## New research provides evidence for breast cancer metabolic adaptation to the unique brain microenvironment

A recent elegant study by Neman and colleagues [15] investigated whether human BCBMs develop from primed cells in the primary tumor, or if surviving metastatic cells adapt to the brain’s unique microenvironment. The authors compare triple-negative and HER2+ primary breast tumors, brain metastases, and representative human-derived cell lines. Relative to both primary tumors and cell lines, the brain metastases exhibit increased expression of several components related to signaling of the inhibitory neurotransmitter γ-aminobutyric acid (GABA), including GABA_A_ receptors, GABA transporters (vesicular GABA transporter, GABA transporter 1-3, GABA-betaine transporter, and glutamate decarboxylase), and GABA transaminase. The study demonstrates that upregulation of GABA-related proteins enables increased transport and metabolism of GABA in BCBMs cells, thereby increasing cell proliferation. The BCBMs cells also acquire features of GABAergic interneurons, expressing interneuron-specific markers like parvalbumin and reelin, characteristics not seen in the primary tumors.

## Future treatments should target the permissive brain microenvironment and the resulting adaptations in addition to breast cancer cells themselves

This work represents one of the few studies that directly compares human primary breast tumors with brain metastases (rather than assessing brain metastases alone and/or breast cancer cell lines *in vitro* or in mouse models) to identify novel factors involved in BCBM biology. These results also add to a growing body of evidence showing a ‘breast-to-brain’ transition with cancer cells adapting to the unique brain microenvironment and acquiring neuronal characteristics that are not seen at either the primary and/or other non-brain metastatic sites [16]. The possibility remains, however, that a small population of ‘seed’ cells in primary tumors are primed for brain metastases with additional adaptations that occur in the brain microenvironment ‘soil’ [17,18].

The acquisition of a novel, neuronal-like GABA shunt metabolism by the BCBM cells is one of the most groundbreaking findings of Neman and colleagues’ article. Cancer cells can exhibit a glycolytic, Warburg-like metabolism instead of oxidative phosphorylation as seen in normal tissues [19]; the GABA shunt in BCBMs reflects yet another metabolic and proliferative advantage utilized by breast cancer cells within the neural environment by providing additional energy through generation of nicotinamide adenine dinucleotide. This acquired metabolism may provide a unique target for brain metastases using therapies that inhibit GABA metabolism. Notably, several inhibitors of GABA are approved in the clinical setting for treatment of epilepsy [20]; however, careful research is required to determine whether selectively targeting this oncometabolite in the setting of BCBM will be safe and effective. Additionally, *in vivo* modeling of GABA metabolism in BCBMs is needed to further establish this adaptive response in the context of a full organism. Ultimately, the work presented by Neman and colleagues places us one step closer to successful therapeutic strategies targeting not only breast cancer cells themselves, but also the supporting brain microenvironment, with the goal of improving survival for patients with BCBM.

## Abbreviations

BCBM: Breast cancer brain metastasis; GABA: γ-aminobutyric acid.

## Competing interests

The authors declare that they have no competing interests.

## Authors’ contributions

All authors contributed to the writing and editing of this manuscript.
